# SMAGEXP: a galaxy tool suite for transcriptomics data meta-analysis

**DOI:** 10.1093/gigascience/giy167

**Published:** 2019-01-29

**Authors:** Samuel Blanck, Guillemette Marot

**Affiliations:** 1 Univ. Lille, CHU Lille , EA 2694 CERIM, 1 place de Verdun, F-59000 Lille, France; 2 Inria Lille-Nord Europe, MODAL, 40 avenue Halley, 59650 Villeneuve d'Ascq , France

**Keywords:** galaxy, transcriptomics, microarray, RNA-seq, meta-analysis

## Abstract

**Background:**

With the proliferation of available microarray and high-throughput sequencing experiments in the public domain, the use of meta-analysis methods increases. In these experiments, where the sample size is often limited, meta-analysis offers the possibility to considerably enhance the statistical power and give more accurate results. For those purposes, it combines either effect sizes or results of single studies in an appropriate manner. R packages metaMA and metaRNASeq perform meta-analysis on microarray and next generation sequencing (NGS) data, respectively. They are not interchangeable as they rely on statistical modeling specific to each technology.

**Results:**

SMAGEXP (Statistical Meta-Analysis for Gene EXPression) integrates metaMA and metaRNAseq packages into Galaxy. We aim to propose a unified way to carry out meta-analysis of gene expression data, while taking care of their specificities. We have developed this tool suite to analyze microarray data from the Gene Expression Omnibus database or custom data from Affymetrix^©^ microarrays. These data are then combined to carry out meta-analysis using metaMA package. SMAGEXP also offers to combine raw read counts from NGS experiments using DESeq2 and metaRNASeq package. In both cases, key values, independent from the technology type, are reported to judge the quality of the meta-analysis. These tools are available on the Galaxy main tool shed. A dockerized instance of galaxy containing SMAGEXP and its dependencies is available on Docker hub. Source code, help, and installation instructions are available on GitHub.

**Conclusion:**

The use of Galaxy offers an easy-to-use gene expression meta-analysis tool suite based on the metaMA and metaRNASeq packages.

## Background

Meta-analyses are widely used in medicine and health policy to increase statistical power in studies suffering from small sample sizes. Gene expression experiments are a typical example of such designs. The R packages metaMA and metaRNASeq are dedicated to gene expression microarray and next-generation sequencing (NGS) meta-analysis, respectively. While metaMA and metaRNASeq are open source and available on CRAN, they require coding skills in R to perform meta-analysis. Thus, to facilitate the use and the dissemination of these packages, we developed Galaxy wrappers. Galaxy [1–3] is an open, web-based platform for data-intensive biomedical research. It keeps tracks of history, and all analyses can be rerun. The Galaxy community is very active, and numerous bioinformatics tools are included in Galaxy thanks to a modular system based on XML wrappers. These integrated tools can be shared via the Galaxy toolshed, which serves as an app store.

## Methods

### Overview of R packages integrated into Galaxy

#### metaMA

Gene expression microarray data meta-analysis can be performed thanks to the metaMA [4] R package. It proposes methods to combine either *P* values or moderated effect sizes from different studies to find differentially expressed (DE) genes. In our pipeline we only keep the inverse normal method [5] to combine the *P*values calculated by limma [6] for each single study.

#### metaRNAseq

RNA sequencing (RNA-seq) data meta-analysis can be performed thanks to the metaRNASeq [7] R package. It implements two *P* value combination techniques: the inverse normal and Fisher methods [8]. Single study *P* values are computed with DESeq2 [9].

#### Differences between metaMA and metaRNASeq

Main differences come from the statistical distributions used to model data and from the manner to treat the genes exhibiting conflicting expression patterns (i.e., under-expression when comparing one condition to another in one study, and over-expression for the same comparison in another study). Usually, microarray data are modeled by Gaussian distributions, while NGS data are modeled by negative binomial distributions. As explained in [4] and [7], the trick to use one-tailed *P* values for each single study before combination in metaMA avoids directional conflicts. In metaRNASeq, this trick cannot be used, which necessitates a *post hoc* identification of conflicts, a step that is also proposed in metaRNASeq.

### Description of Galaxy tools

**Figure 1:**
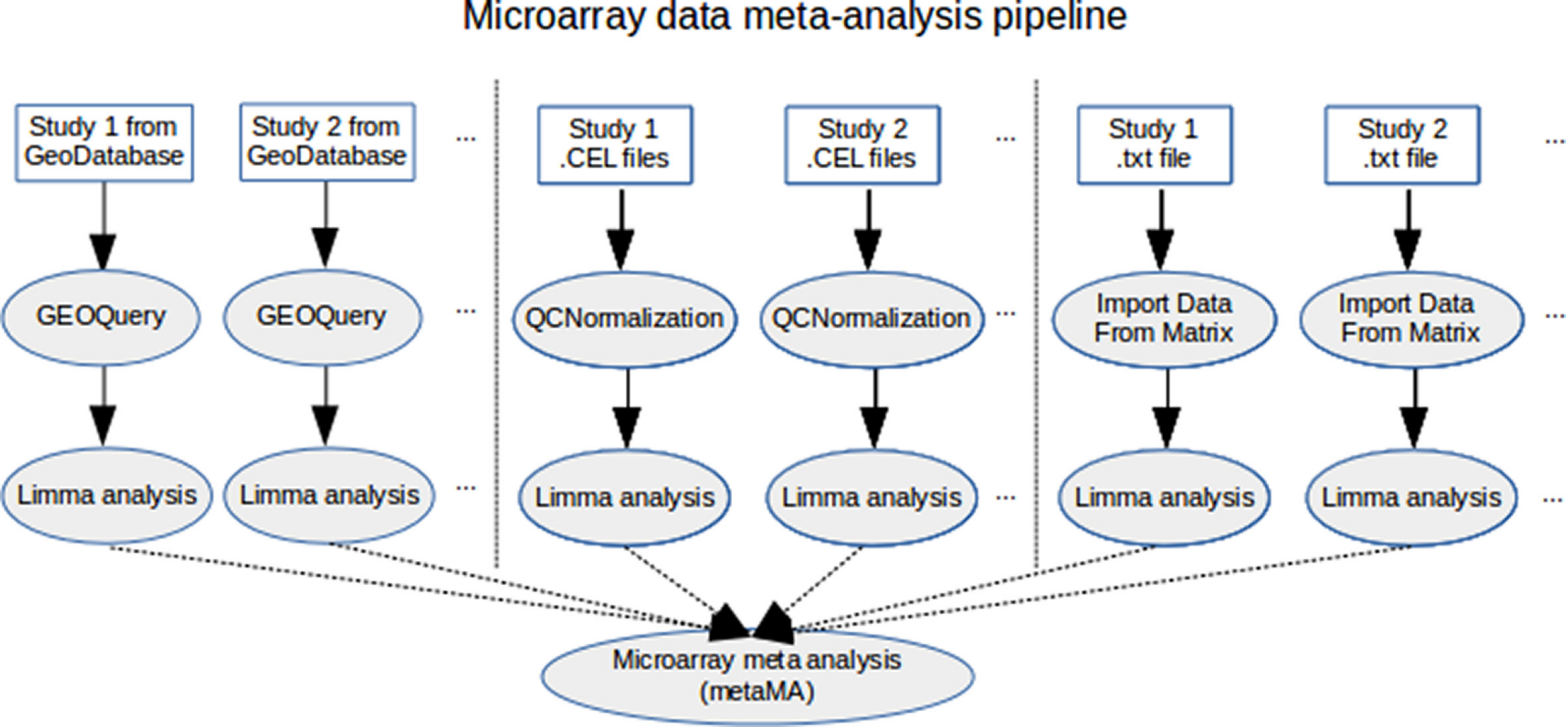
Overview of the tools from microarray data meta-analysis pipeline integrated within Galaxy.

The SMAGEXP tool suite offers two distinct gene expression meta-analysis functionalities: one dedicated to microarray data meta-analysis and one dedicated to RNA-seq data meta-analysis (see Table 1 and Fig. 1).

**Table 1:** Summary of tool inputs and outputs

Tool	Input	Output
GEOQuery	Gene Expression Omnibus database ID	rdata object and .cond file
QCNormalization	Raw .CEL Affymetrix^©^ files	rdata object and plots
Import custom data	Expression data in tabular text format	rdata object and plots
Limma analysis	rdata object from GEOQuery or QCNormalization or Import custom data and .cond file	rdata Object, HTML report and results text file
Microarray data meta-analysis	rdata objects from Limma analyses	HTML report
Recount	Recount accession ID	One count file per sample
RNA-seq data meta-analysis	Results text files from galaxy DESeq2 tool	HTML report

#### Microarray data meta-analysis

##### GEOQuery tool.

**Figure 2:**
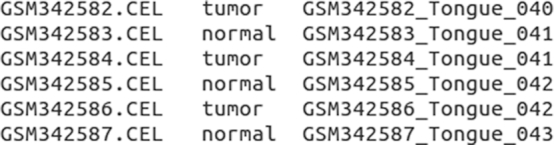
Example of .cond file.

GEOQuery tool fetches microarray data directly from Gene Expression Omnibus (GEO) database [10], based on the GEOQuery [11] bioconductor [12] R package. Given a GSE accession ID, it returns an rdata object containing the data and a text file (.cond file, see Fig. 2) summarizing the conditions of the experiment. The .cond file is a text file containing one line per sample in the experiment. Each line is made of 3 columns:

- Sample ID
- Condition of the biological sample
- Description of the biological sample

Column names are optional, and only the columns order matters. As the GEO dataset should already have been normalized, the GEOQuery tool does not perform any normalization method, apart from an optional log2 transformation.

##### QCNormalization tool.

It is possible to analyze .CEL files from Affymetrix^©^ gene expression microarray. The QCnormalization tool offers to ensure the quality of the data and to normalize them. Several normalization methods are available:

- rma normalization
- quantile normalization + log2
- background correction + log2
- log2 only

This tool generates several quality figures: microarray images, box plots, and MA plots. It also outputs an rdata object containing the normalized data for further analysis with the limma analysis tool.

##### Import custom data tool.

This tool imports data stored in a tabular text file. A few normalization methods are proposed, but it is possible to skip the normalization step by choosing “none” in the normalization methods options. Therefore, this tool is of special interest when the input dataset has been previously normalized. This tool also generates box plots and MA plots and outputs an rdata object containing the data for further analysis with the limma analysis tool.

##### Limma analysis tool.

**Figure 3:**
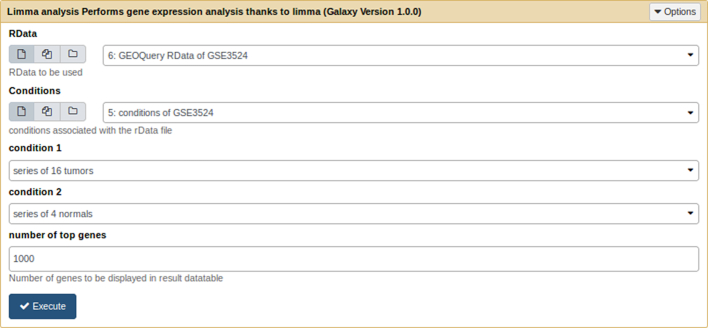
limma analysis tool form.

The Limma analysis tool performs single analysis either of data previously retrieved from the GEO database or normalized Affymetrix^©^ .CEL files data. Given a .cond file, it runs a standard limma differential expression analysis. The user choose two conditions extracted from the .cond file (see Fig. 3). It generates box plots for rough quality control of normalization, *P* value histograms to ensure that statistical hypotheses are not violated, and a volcano plot to quickly identify the most meaningful changes. This tool also outputs a table summarizing the DE genes and their annotations. Genes are sorted by ascending Benjamini-Hochberg adjusted *P* value, and annotations are retrieved via GEO database. This list of genes can be exported to excel or to csv format. This table is sortable and requestable. Furthermore, it is possible to expand each row to display extended annotation information, including hypertext links to the National Center for Biotechnology Information (NCBI) gene database. Finally, this tool outputs an rdata object to perform further meta-analysis and a text file containing annotated results of the differential analysis.

##### Microarray data meta-analysis tool.

The meta-analysis relies on the metaMA R package. Prior to the meta-analysis itself, a pre-processing is made in order to ensure compatibility between several sources of data. In fact, data could come from different types of microarrays. First, we list the Entrez gene ID corresponding to each probe of each dataset. Next, we keep the probes corresponding to the genes that are shared by all the experiments of the meta-analysis. Then, for each dataset, we merge the microarray probes originating from the same Entrez gene ID by computing their mean. Note that the merging of different technologies induces a loss of information and might generate several conflicts as probes do not necessarily reflect the same biological reality. Finally, the *P* value combination method of metaMA is run on the merged dataset. It generates a Venn diagram (if the number of studies is lower than 3) or a UpSet diagram [13] (if the number of studies is greater than 4 ) summarizing the results of the meta-analysis, and a list of indicators to evaluate the quality of the performance of the meta-analysis:

- DE (differentially expressed): number of DE genes
- IDD (integration-driven discoveries): number of genes that are declared DE in the meta-analysis that were not identified in any of the single studies alone
- Loss: number of genes that are identified DE in single studies but not in meta-analysis
- IDR (integration-driven discovery rate): corresponding proportion of IDD
- IRR (integration-driven revision): corresponding proportion of loss

It also outputs a fully sortable and requestable table, with gene annotations and hypertext links to NCBI gene database.

#### RNA-seq data meta-analysis

##### Recount tool.

The recount tool fetches data from the recount2 project database [14]. The recount Galaxy tool relies on the bioconductor R package recount. Given the accession ID of an experiment, it generates one count file per sample of the experiment. Then these files can be analyzed by the Galaxy DESeq2 tool.

##### RNA-seq data meta-analysis tool.

The RNA-seq data meta-analysis tool relies on the DESeq2 galaxy tool analysis results. Given several text files resulting from the DESeq2 [9] tool, the metaRNAseq tool performs a meta-analysis, generates the list of DE genes, and outputs the DE, IDD, loss, IDR, and IRR indicators.

## Application

### Microarray meta-analysis example

**Figure 4:**
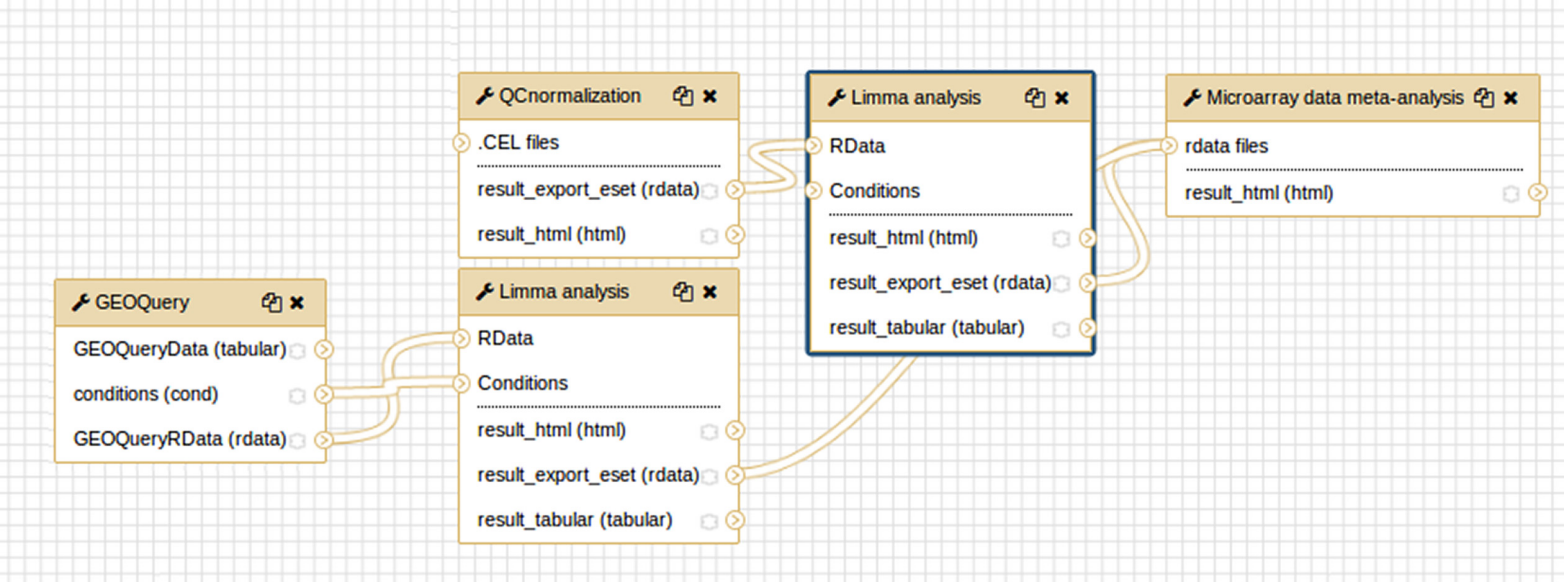
Exemple of a galaxy workflow for microarray meta-analysis.

SMAGEXP was applied to two GEO datasets identified with the following IDs: GSE3524 [15] and GSE13601 [16]. These two datasets contain human oral squamous cell carcinoma (SCC) data. See Fig. 4 for an overview of the worfklow of this analysis.

**Figure 5:**
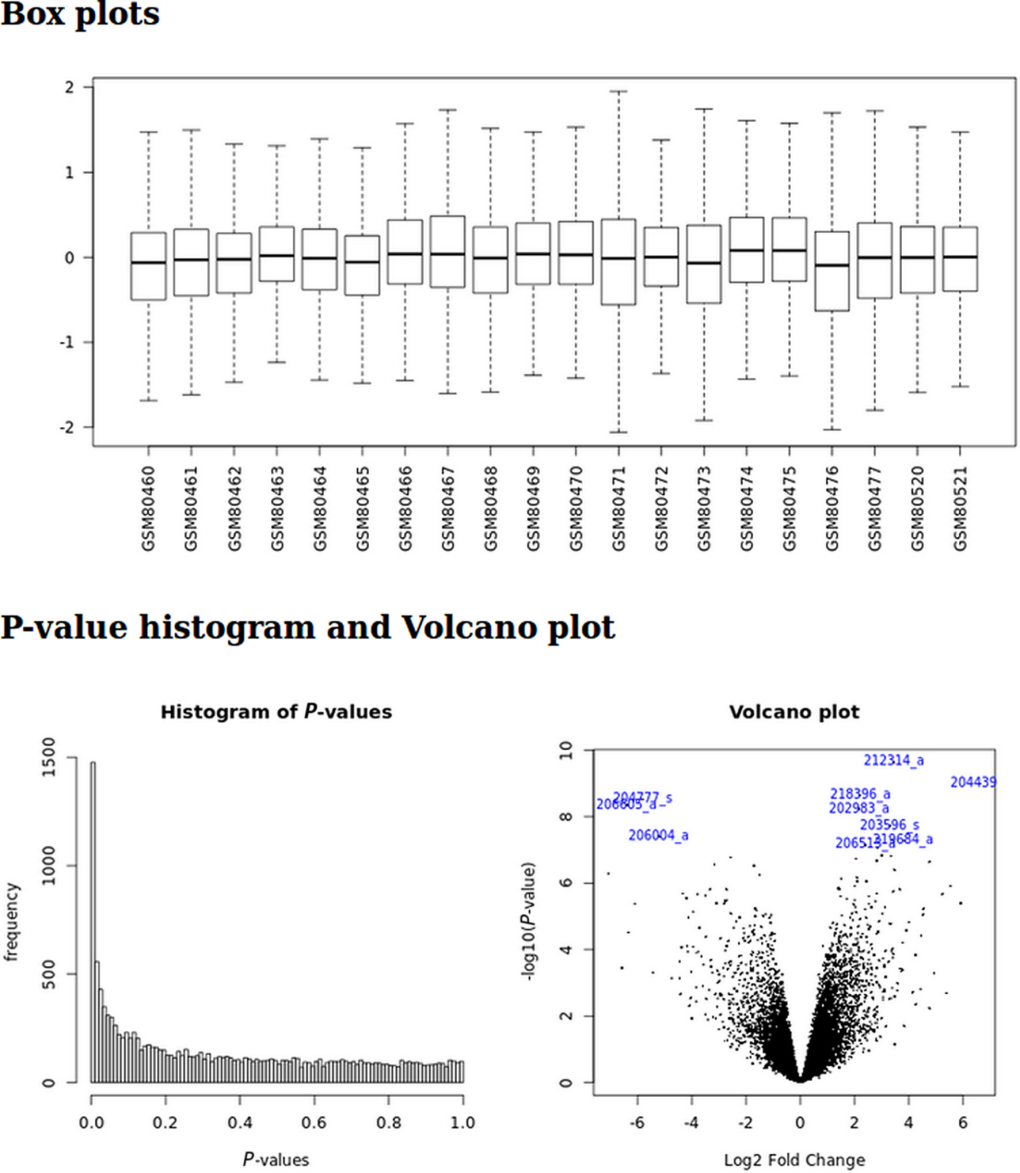
limma analysis tool output plots.

**Figure 6:**
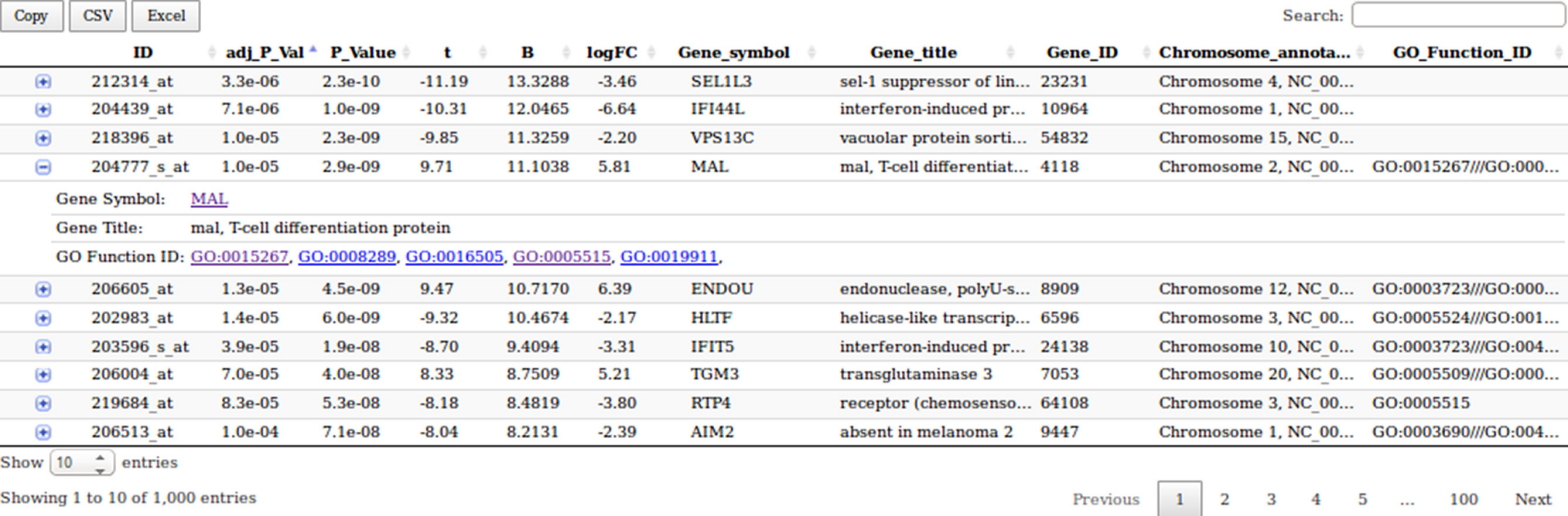
limma analysis tool: table of top 10 genes for GSE3524 dataset.

First, we fetch data from the GSE3524 using the GEOQuery tool (with parameter “log2 transformation” = auto). Then, we launch the limma analysis, using the output from the GEOquery tool. It generates an rdata output that will be useful for the meta-analysis. Results can be seen in Figs. 5 and 6

Secondly, the same kind of analysis is run from raw .CEL files. We choose to keep six .CEL files from the GSE13601 dataset (IDs from GSM342582 to GSM342587). Quality control and normalization are done thanks to the QCnormalization tool. Then, as previously, the limma analysis tool is run to generate an HTML report and an rdata output.

#### Run a metaMA analysis

**Figure 7:**
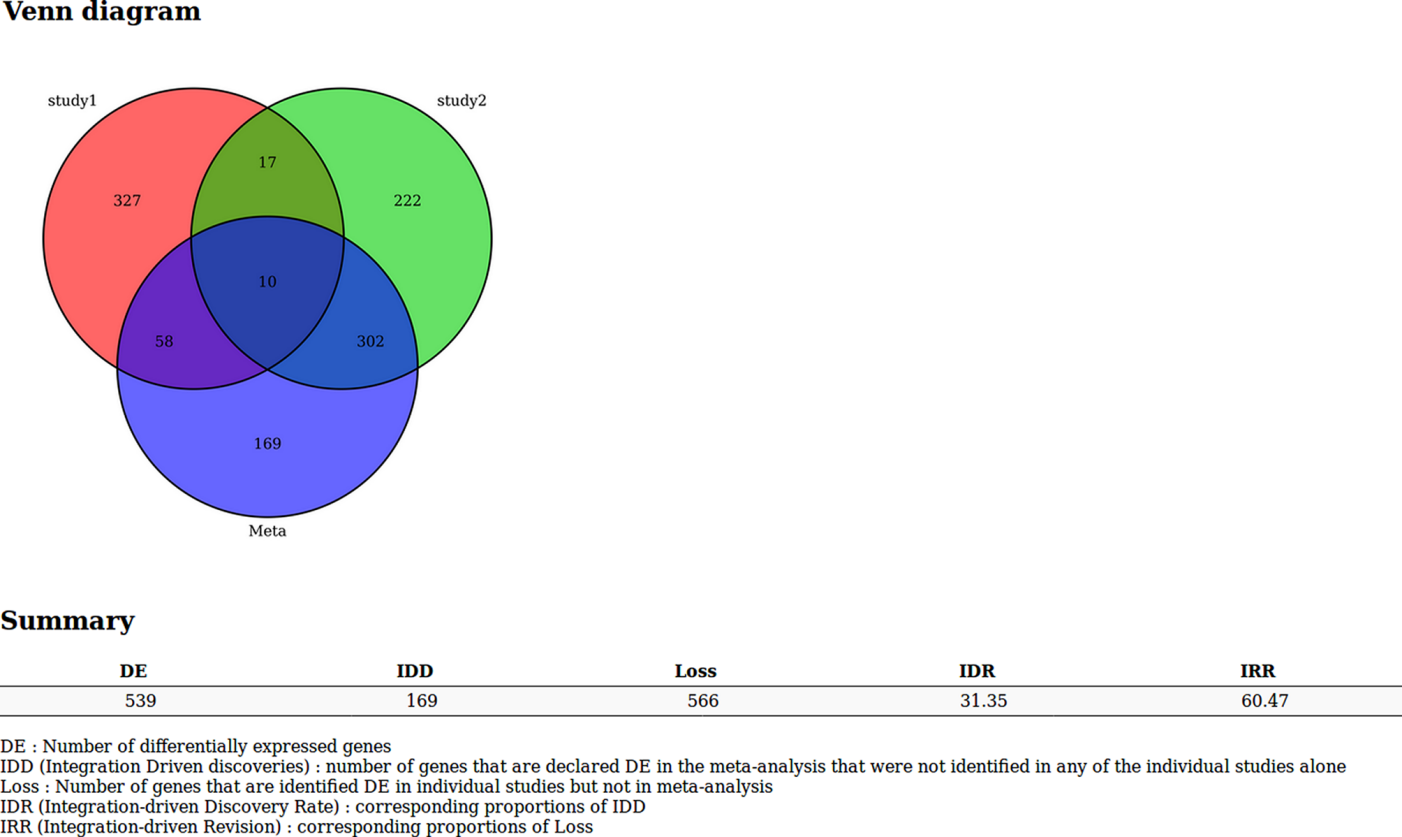
Venn diagram and summary of microarray data meta-analysis tool results.

To run the microarray meta-analysis tool, we only need the rdata output of each single study, generated by the limma analysis tool. It generates a Venn diagram or an UpSet plot (when the number of studies is greater than 3) to compare the results of each study with the meta-analysis. It also outputs several indicators as described in the description of the tool (see Fig. 7). As for the limma tool, annotated expressed genes are displayed in a table that can be ordered and requested.

### RNA-seq data meta-analysis example

SMAGEXP was applied to three Recount2 datasets identified with the following IDs: SRP032833 [17], SRP028180 [18], and SRP058237 [19]. These three datasets contain human lung SCC data. We first fetch data from these datasets with the recount galaxy tool. Then, thanks to the Galaxy DESeq2 tool, we launch differential analysis on the following contrasts: invasive vs normal for SRP032833 dataset, tumor vs normal for SRP028180 dataset, and tumor vs adjacent for SRP058237 dataset.

#### Run a metaRNAseq analysis

The RNA-seq data meta-analysis tool relies on DESeq2 results

**Figure 8:**
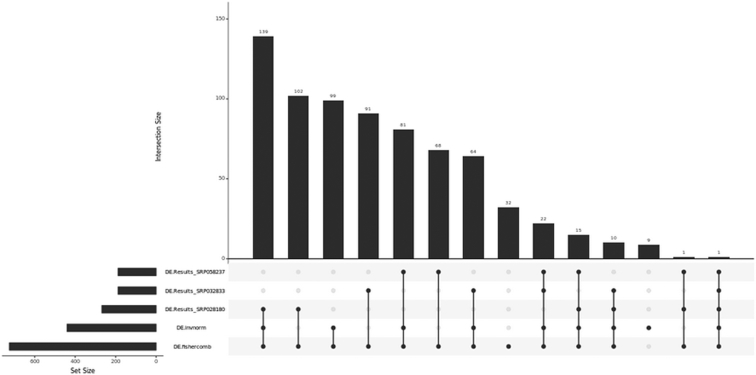
UpSet plot for the RNA-seq datasets SRP032833, SRP028180, and SRP058237.

**Figure 9:**
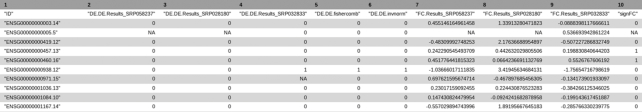
Header of a metaRNAseq results file.

It outputs a Venn diagram or an UpSet plot (if the number of studies is greater than 3, see Fig. 8) and the same indicators as in the microarray data analysis tool for both Fisher and inverse normal *P* values combinations. It also generates a text file containing summarization of the results of each single analysis and meta-analysis. Potential conflicts between single analysis are indicated by zero values in the “signFC” column (see Fig. 9).

## Conclusion

We developed SMAGEXP, a tool suite dedicated to gene-expression data meta-analysis. This tool suite proposes quality controls, single analyses, and meta-analyses of microarray and RNA-seq data, suggesting appropriate pipelines for each type of data. It delivers fully annotated results of differentially DE genes, exportable in several usual formats. Integrated into Galaxy, SMAGEXP is easy to use for biologists and life scientists. R packages metaMA and metaRNAseq thus inherit reproducibility and accessibility support from Galaxy. Furthermore, thanks to Docker, we made these Galaxy tools and their dependencies easy to deploy.

## Availability of source code and requirements

- Project name: SMAGEXP
- Project home page: https://github.com/sblanck/smagexp [20]
- Operating system(s): Linux (Galaxy); platform independent for Galaxy’s browser-based user interface.
- Programming language: R
- Other requirements: Galaxy, Docker [21]
- License: MIT license
- Any restrictions to use by non-academics: None
- SciCrunch.org RRID:SCR_016360

SMAGEXP is available on the Galaxy main toolshed [22]. Furthermore, a fully dockerized instance of Galaxy containing SMAGEXP and DESeq2 is available at: https://hub.docker.com/r/sblanck/galaxy-smagexp/.

## Availability of supporting data

The datasets supporting the microarray meta-analysis example presented here are available in the GEO database. Their accession IDs are GSE3524 and GSE13601. The datasets supporting the RNA-seq meta-analysis example presented here are available on Recount2. Their accession IDs are SRP032833, SRP028180, and SRP058237

Documentation, step-by-step tutorials, examples, galaxy histories, and workflow presented here are available on GitHub: https://github.com/sblanck/smagexp/tree/master/examples.

Code snapshots and input data are available from the GigaScience GigaDB repository [23].

## Abbreviations

DE, differentially expressed; GEO, Gene Expression Omnibus; IDD, integration-driven discoveries; IDR, integration-driven discovery rate; IRR, integration-driven revision; NCBI, National Center for Biotechnology Information; NGS, next-generation sequencing; RNA-seq, RNA sequencing; SCC, squamous cell carcinoma; SMAGEXP, Statistical Meta-Analysis for Gene EXPression.

## Competing interests

The authors declare that they have no competing interests.

## Author contributions

The project was initiated by G.M. who developed metaMA and metaRNASeq R packages. The galaxy tools were developed, installed, and documented by S.B. and tested by S.B. and G.M. The article was written by S.B. and G.M. Both authors read and approved the final manuscript.

## Supplementary Material

GIGA-D-18-00078_Original_Submission.pdf

GIGA-D-18-00078_Revision_1.pdf

GIGA-D-18-00078_Revision_2.pdf

GIGA-D-18-00078_Revision_3.pdf

Response_to_Reviewer_Comments_Original_Submission.pdf

Response_to_Reviewer_Comments_Revision_1.pdf

Response_to_Reviewer_Comments_Revision_2.pdf

Reviewer_1_Report_Original_Submission -- Kieran O'Neill 3/21/2018 Reviewed

Reviewer_1_Report_Revision_1 -- Kieran O'Neill 7/25/2018 Reviewed

Reviewer_2_Report_Original_Submission -- Nitesh Turaga 3/29/2018 Reviewed
