# Supplementary material for: SMAGEXP: a galaxy tool suite for transcriptomics data meta-analysis
Source: Gigascience. 2019 Jan 29;8(2):giy167. doi: 10.1093/gigascience/giy167 (PMC6354025; doi:10.1093/gigascience/giy167)
Supplement: GIGA-D-18-00078_Revision_1.pdf [file giy167_giga-d-18-00078_revision_1.pdf]

GigaScience

SMAGEXP: a galaxy tool suite for transcriptomics data meta-analysis

--Manuscript Draft--

**Manuscript Number:**	GIGA-D-18-00078R1
**Full Title:**	SMAGEXP: a galaxy tool suite for transcriptomics data meta-analysis
**Article Type:**	Technical Note
**Funding Information:**	
**Abstract:**	Background<br>With the proliferation of available microarray and high throughput sequencing experiments in the public domain, the use of meta-analysis methods increases. In these experiments, where the sample size is often limited, meta-analysis offers the possibility to considerably enhance the statistical power and give more accurate results. For those purposes, it combines either effect sizes or results of single studies in a appropriate manner.<br>R packages metaMA and metaRNASeq perform meta-analysis on microarray and NGS data, respectively. They are not interchangeable as they rely on statistical modeling specific to each technology.<br><br>Results<br>SMAGEXP (Statistical Meta-Analysis for Gene EXPression) integrates metaMA and metaRNAseq packages into Galaxy. We aim to propose a unified way to carry out meta-analysis of gene expression data, while taking care of their specificities.<br>We have developed this tool suite to analyse microarray data from Gene Expression Omnibus (GEO) database or custom data from affymetrix microarrays. These data are then combined to carry out meta-analysis using metaMA package. SMAGEXP also offers to combine raw read counts from Next Generation Sequencing (NGS) experiments using DESeq2 and metaRNASeq package. In both cases, key values, independent from the technology type, are reported to judge the quality of the meta-analysis. These tools are available on the Galaxy main tool shed. Source code, help and installation instructions are available on github.<br><br>Conclusion<br>The use of Galaxy offers an easy-to-use gene expression meta-analysis tool suite based on the metaMA and metaRNASeq packages.
**Corresponding Author:**	samuel blanck, M.D.<br>Universite Lille 2 Droit et Sante<br>LILLE, FRANCE
**Corresponding Author Secondary Information:**	
**Corresponding Author's Institution:**	Universite Lille 2 Droit et Sante
**Corresponding Author's Secondary Institution:**	
**First Author:**	Samuel Blanck
**First Author Secondary Information:**	
**Order of Authors:**	Samuel Blanck
	Guillemette Marot
**Order of Authors Secondary Information:**	
**Response to Reviewers:**	We thank the Editor and the two Reviewers for their comments and suggestions to improve the quality of the paper. We have now taken into account all the points raised in<br>the reviews.<br><br>Reply to editor :

SMAGEXP has been registered in the SciCrunch.org database with the RRID SCR:016360. It is included in the Availability of source code and requirements section

Reply to reviewer 1

1. We mentionned it in the results part of the abstract, in the conclusion of the manuscript and in the availability of source code and requirements.

2.a The GEOquery bug is fixed.

2.b We did not find an easy way to include histories inside the docker image. Nevertheless, all the histories, data, and workflow are available on github at https://github.com/sblanck/smagexp/tree/master/examples and can easily, once logged in Galaxy, be imported into the dockerized version of Galaxy.

3. UpSet plots have been added. We chose to keep Venn diagram when the number of studies is <=3 for micro-array and =2 for RNA-seq (since two p-value combination methods are available for this technology). If the number of studies is greater, we plot UpSet diagrams.

4.a A step by step tutorial with screenshots was added in the github documentation

4.b The inline documentation of RNA-seq meta-analysis was added.

4.c This mention was added in the documentation on github.

4.d The .cond file is not needed any more with the import custom data tool. Nevertheless, it is still required for the limma analysis. The format of the .cond file is described in the documentation and examples of .cond file are available on github.

4.e This instruction was added in the documentation.

5. It is not possible to easily retrieve RNA-seq count data with GEOquery from Galaxy. The count files are sometimes available as supplementary data, but the format often varies from one dataset to another one.
We propose to fetch raw count data from recount2 project. We developped a wrapper to retrieve data from recount2 and generate count files. These count files are suitable for the galaxy DESeq2 tool which was added to the docker image alongside SMAGEXP. Note that recount always outputs the same number of transcripts for a given species. Therefore we have an easy way to meta-analyze rna-seq data from real world.

6. The NAR paper assumes that data coming from micro-array an rna-seq are similar as they calculate their effect sizes the same way. In particular, the calculation described is relevant for Gaussian data, questionnable for count data. As we already mentioned in "Rau et~al.(2014), Differential meta-analysis of RNA-seq data from multiple studies, BMC Bioinformatics 2014;15(1):1--10" , we believe that the Gaussian distribution is not adapted for most RNA-Seq datasets, without transformation. Several transformations have been suggested in the litterature but the automatic choice of the better one is still a question of research for statisticians so we prefer that users treat RNAseq and microarray data seperately, even if they can merge the results via VennDiagrams.

Reply to reviewer 2

1. Docker hub link has been corrected

2. The rdata objects contain Bioconductor ExpressionSet objects, containing expression data and meta-data of the experiments. Their content is described on the github page. They are given for people who are familiar with the R programming language and can be directly loaded in rStudio environment.
It seems difficult to have an interaction between Galaxy and rStudio in the docker container and we believe that people who use Galaxy do not need these rdata inside

Powered by Editorial Manager® and ProduXion Manager® from Aries Systems Corporation

	Galaxy. All the valuable information of these rdata is also available in text format in Galaxy.<br><br>3. The workflow and histories are now available on github and can easily be imported in Galaxy (inside the docker container or on a standalone instance)\\ https://github.com/sblanck/smagexp/tree/master/examples<br><br>minor :<br><br>1. Done<br><br>2. Bioconductor citation has been added.
**Additional Information:**	
**Question**	**Response**
Are you submitting this manuscript to a special series or article collection?	No
**Experimental design and statistics**<br><br>Full details of the experimental design and statistical methods used should be given in the Methods section, as detailed in our Minimum Standards Reporting Checklist. Information essential to interpreting the data presented should be made available in the figure legends.<br><br>Have you included all the information requested in your manuscript?	Yes
**Resources**<br><br>A description of all resources used, including antibodies, cell lines, animals and software tools, with enough information to allow them to be uniquely identified, should be included in the Methods section. Authors are strongly encouraged to cite Research Resource Identifiers (RRIDs) for antibodies, model organisms and tools, where possible.<br><br>Have you included the information requested as detailed in our Minimum Standards Reporting Checklist?	Yes
**Availability of data and materials**	Yes

Powered by Editorial Manager® and ProduXion Manager® from Aries Systems Corporation

All datasets and code on which the conclusions of the paper rely must be either included in your submission or deposited in publicly available repositories (where available and ethically appropriate), referencing such data using a unique identifier in the references and in the “Availability of Data and Materials” section of your manuscript.<br><br>Have you have met the above requirement as detailed in our Minimum Standards Reporting Checklist?	

Manuscript
Click here to access/download;Manuscript;article.tex
Click here to view linked References

Placeholder for OUP logo oup.pdf

Placeholder for journal logo gigascience-logo.pdf

GigaScience, 2018, 1–7

doi: xx.xxxx/xxxx
Manuscript in Preparation
Technical note

TECHNICAL NOTE

SMAGEXP: a galaxy tool suite for transcriptomics data meta-analysis

Samuel Blanck[1,*] and Guillemette Marot[2]

[1]Univ. Lille Droit et Santé, EA 2694, F-59000 Lille, France and [2]Inria Lille-Nord Europe, MODAL, F-59000 Lille, France

[*]samuel.blanck@univ-lille.fr

Abstract

With the proliferation of available microarray and high throughput sequencing experiments in the public domain, the use of meta-analysis methods increases. In these experiments, where the sample size is often limited, meta-analysis offers the possibility to considerably enhance the statistical power and give more accurate results. For those purposes, it combines either effect sizes or results of single studies in a appropriate manner. R packages metaMA and metaRNASeq perform meta-analysis on microarray and NGS data, respectively. They are not interchangeable as they rely on statistical modeling specific to each technology.
Results: SMAGEXP (Statistical Meta-Analysis for Gene EXPression) integrates metaMA and metaRNAseq packages into Galaxy. We aim to propose a unified way to carry out meta-analysis of gene expression data, while taking care of their specificities. We have developed this tool suite to analyse microarray data from Gene Expression Omnibus (GEO) database or custom data from Affymetrix© microarrays. These data are then combined to carry out meta-analysis using metaMA package. SMAGEXP also offers to combine raw read counts from Next Generation Sequencing (NGS) experiments using DESeq2 and metaRNASeq package. In both cases, key values, independent from the technology type, are reported to judge the quality of the meta-analysis. These tools are available on the Galaxy main tool shed. A dockerized instance of galaxy containing SMAGEXP and its dependencies is available on Docker hub. Source code, help and installation instructions are available on github.
Conclusion: The use of Galaxy offers an easy-to-use gene expression meta-analysis tool suite based on the metaMA and metaRNASeq packages.

Key words: Galaxy; Transcriptomics; microarray ; RNA-seq; Meta-analysis

Background

Meta-analyses are widely used in medicine and health policy to increase statistical power in studies suffering from small sample sizes. Gene expression experiments are a typical example of such designs. The R packages metaMA and metaRNASeq are dedicated to gene expression microarray and NGS meta-analysis, respectively. While metaMA and metaRNASeq are open source and available on CRAN, they require coding skills in R to perform meta-analysis. Thus, to facilitate the use and the dissemination of these packages, we developed Galaxy wrappers. Galaxy [1, 2, 3] is an open, web-based platform for data intensive biomedical research. It keeps tracks of history and all analyses can be rerun. Galaxy community is very active and a lot of bioinformatics tools are included in Galaxy thanks to a modular system based on XML wrappers. These integrated tools can be shared via the Galaxy toolshed which serves as an appstore.

Methods

Compiled on: June 27, 2018.
Draft manuscript prepared by the author.

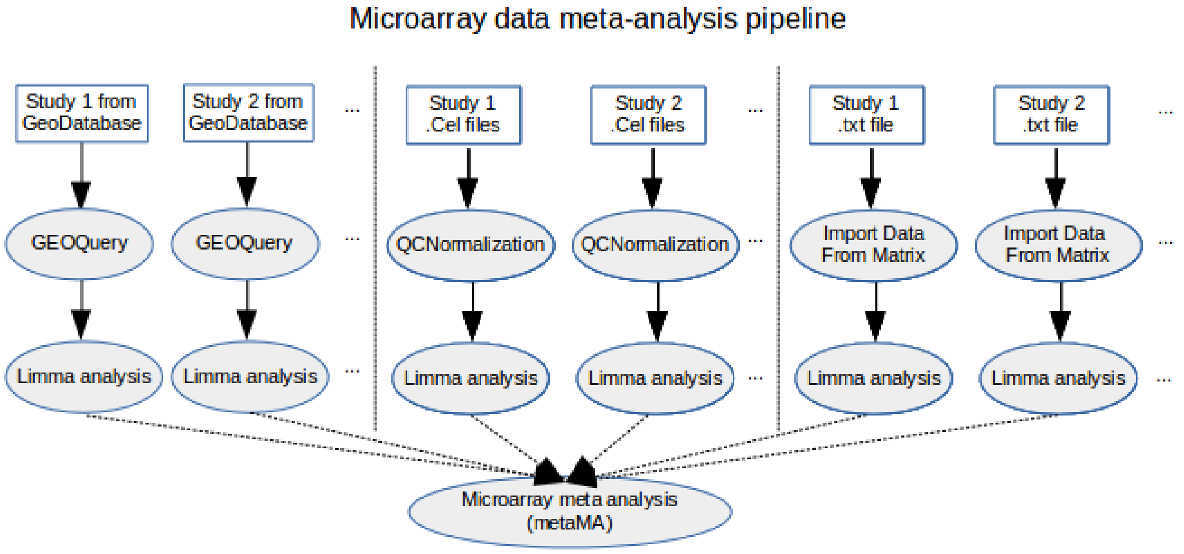

Figure 1. Overview of the tools from microarray data meta-analysis pipeline integrated within Galaxy.

Overview of R packages integrated into Galaxy

metaMA

Gene expression microarray data meta-analysis can be performed thanks to the metaMA [4] R package. It proposes methods to combine either p-values or moderated effect sizes from different studies to find differentially expressed genes. In our pipeline we only keep the inverse normal method [5] to combine the p-values calculated by limma [6] for each single study.

metaRNAseq

RNA-seq data meta-analysis can be performed thanks to the metaRNASeq [7] R package. It implements two p-value combination techniques : the inverse normal and Fisher methods [8]. Single study p-values are comptuted with DESeq2 [9].

Differences between metaMA and metaRNASeq

Main differences come from the statistical distributions used to model data and from the manner to treat the genes exhibiting conflicting expression patterns (i.e., under-expression when comparing one condition to another in one study, and over-expression for the same comparison in another study). Usually, microarray data are modelled by Gaussian distributions while NGS data are modelled by Negative Binomial distributions. As explained in [4] and [7], the trick which consists to use one-tailed p-values for each single study before combination in metaMA avoids directional conflicts. In metaRNASeq, this trick can not be used, which necessits a post-hoc identification of conflicts, step which is also proposed in metaRNASeq.

Description of Galaxy tools

SMAGEXP tool suite offers two distinct gene expression meta-analysis functionalities : one dedicated to microarray data meta-analysis and one dedicated to RNAseq data meta-analysis (see Table 1 and figure 1).

Microarray data meta-analysis

GEOQuery tool. GEOQuery tool fetches microarray data directly from GEO database [10], based on the GEOQuery [11] bioconductor [12] R package. Given a GSE accession ID, it returns an rdata object containing the data and a text file (.cond file) summarizing the conditions of the experiment. The .cond file is a text file containing one line per sample in the experiment. Each line is made of 3 columns:

- Sample ID
- Condition of the biological sample
- Description of the biological sample

Column names are optional and only the columns order matters. As the GEO dataset should already have been normalized, the GEOQuery tool does not perform any normalization method, apart from an optional log2 transformation.

QCNormalization tool. It is possible to analyze .CEL files from Affymetrix© gene expression microarray. The QCnormalization tool offers to ensure the quality of the data and to normalize them. Several normalization methods are available :

- rma normalization
- quantile normalization + log2
- background correction + log2
- log2 only

This tool generates several quality figures : microarray images, boxplots and MA plots. It also outputs an rdata object containing the normalized data for further analysis with the limma analysis tool.

Import custom data tool. This tool imports data stored in a tabular text file. A few normalization methods are proposed, but it is possible to skip the normalization step, by choosing "none" in the normalization methods options. Therefore this tool is of special interest when the input dataset has been previously normalized.

This tool also generates boxplots and MA plots and outputs an rdata object containing the data for further analysis with

Table 1. Summary of tools inputs and outputs.

Tool	Input	Output
GEOQuery	GEO database ID	rdata object and .cond file
QCNormalization	Raw .CEL Affymetrix© files	rdata object and plots
Import custom data	Expression data in tabular text format	rdata object and plots
Limma analysis	rdata object from GEOQuery or QCNormalization or Import custom data and .cond file	rdata Object, HTML report and results text file
Microarray data meta-analysis	rdata objects from Limma analyse	HTML report
Recount	Recount accession ID	One count file per sample
RNA-seq data meta-analysis	Results text files from galaxy DESeq2 tool	HTML report

```
GSM342582.CEL   tumor   GSM342582_Tongue_040
GSM342583.CEL   normal  GSM342583_Tongue_041
GSM342584.CEL   tumor   GSM342584_Tongue_041
GSM342585.CEL   normal  GSM342585_Tongue_042
GSM342586.CEL   tumor   GSM342586_Tongue_042
GSM342587.CEL   normal  GSM342587_Tongue_043
```

Figure 2. Example of .cond file.

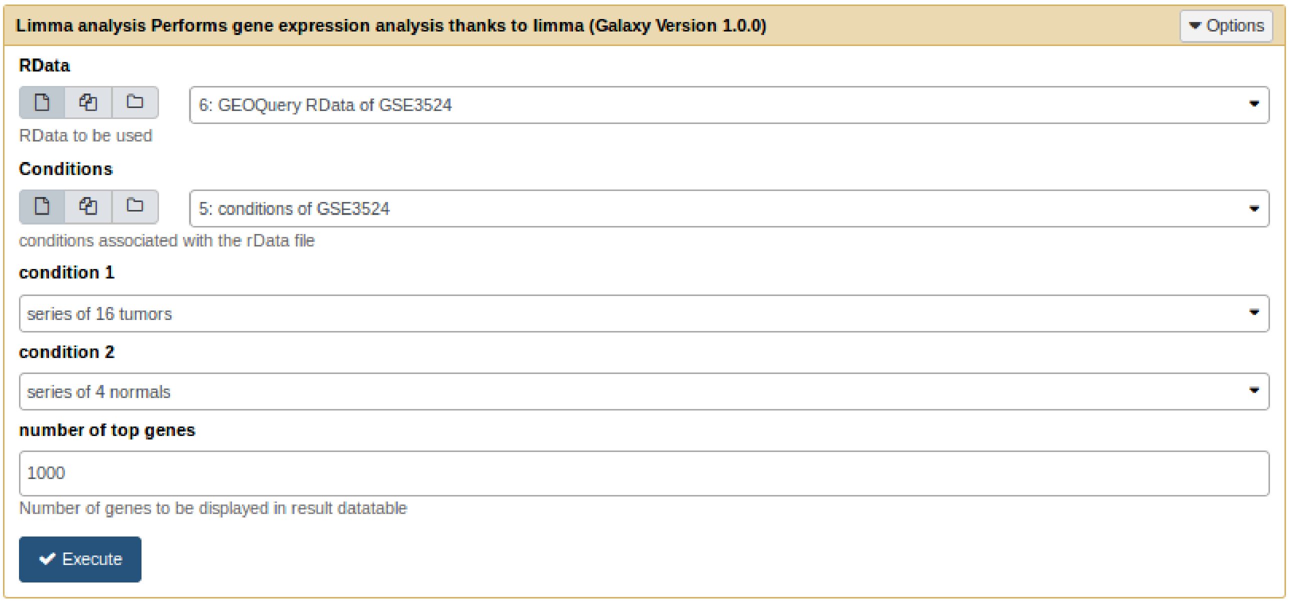

Figure 3. limma analysis tool form.

the limma analysis tool.

Limma analysis tool. The Limma analysis tool performs single analysis either of data previously retrieved from GEO database or normalized Affymetrix© .CEL files data. Given a .cond file, it runs a standard limma differential expression analysis.The user choose two conditions extracted from the .cond file (see Figure 3). It generates boxplots for rough quality control of normalization, p-value histograms to ensure that statistical hypotheses are not violated and a volcano plot to quickly identify the most-meaningful changes. This tool also outputs a table summarizing the differentially expressed genes and their annotations. Genes are sorted by ascending Benjamini-Hochberg adjusted p-value, and annotations are retrieved via GEO database. This list of genes can be exported to excel or to csv format. This table is sortable and requestable. Furthermore it is possible to expand each row to display extended annotations informations, including hypertext links to the National Center for Biotechnology Information (NCBI) gene database. Finally, this tool outputs an rdata object to perform further meta-analysis and a text file containing annotated results of the differential analysis.

Microarray data meta-analysis tool. The meta-analysis relies on the metaMA R package. Prior to the meta-analysis itself, a pre-processing is made in order to ensure compatiblity between several sources of data. In fact, data could come from different types of microarrays. First, we list the Entrez gene ID corresponding to each probe of each dataset. Next, we keep the probes corresponding to the genes which are shared by all the experiments of the meta-analysis. Then, for each dataset, we merge the microarray probes originating from the same Entrez gene ID by computing their mean. Note that the merging of different technologies induces a loss of information and might generate several conflicts as probes do not necessary reflect the same biological reality. Finally, the p-value combination method of metaMA is run on the merged dataset. It generates a Venn diagram (if the number of study in lower or equal than 3) or a UpSet diagram [13] (if the number of studies is greater than 3) summarizing the results of the meta-analysis, and a list of indicators to evaluate the quality of the performance of the meta-analysis :

- DE : Number of differentially expressed genes
- IDD (Integration Driven discoveries) : number of genes that are declared differentially expressed in the meta-analysis that were not identified in any of the single studies alone
- Loss : Number of genes that are identified differentially expressed in single studies but not in meta-analysis
- IDR (Integration-driven Discovery Rate) : corresponding proportion of IDD

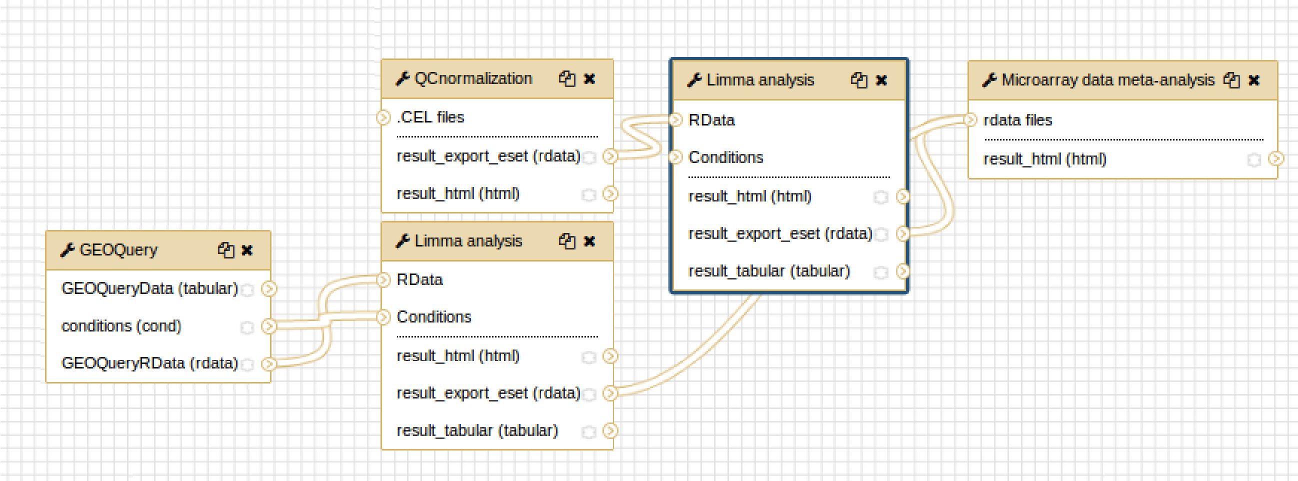

Figure 4. Exemple of a galaxy workflow for microarray meta-analysis.

- IRR (Integration-driven Revision) : corresponding proportion of Loss

It also outputs a fully sortable and requestable table, with gene annotations and hypertext links to NCBI gene database.

RNA-seq data meta-analysis

Recount tool. Recount tool fetches data from the recount2 project database [14]. The recount Galaxy tool rely on the bioconductor R package recount. Given the accession ID of an experiment, it generates one count file per sample of the experiment. Then these files can be analysed by the Galaxy DESeq2 tool.

RNA-seq data meta-analysis tool. The RNA-seq data meta-analysis tool relies on the DESeq2 galaxy tool analysis results. Given several text file resulting from the DESeq2 [9]. tool, the metaRNAseq tool performs a meta-analysis, generates the list of differentially expressed genes, and outputs the DE, IDD, Loss, IDR and IRR indicators.

Application

Microarray meta-analysis example

SMAGEXP was applied to two GEO datasets identified with the following IDs : GSE3524 [15] and GSE13601 [16]. These two datasets contain human oral squamous cell carcinoma (SCC) data. See Figure 4 for an overview of the worfklow of this analysis.

First, we fetch data from the GSE3524 using the GEOQuery tool (with parameter "log2 transformation" = auto). Then we launch the limma analysis, using the output from the GEOquery tool. It generates an rdata output, which will be usefull for the meta-analysis. Results can be seen on Figure 5 and Figure 6

Secondly, the same kind of analysis is run from raw .CEL files. We choose to keep six .CEL files from the GSE13601 dataset (IDs from GSM342582 to GSM342587). Quality control and normalization is done thanks to the QCnormalization tool. Then, as previously, the limma analysis tool is run to generate a HTML report and an rdata output.

Run a metaMA analysis

To run the microarray meta-analysis tool, we only need the rdata output of each single study, generated by the limma analysis tool. It generates a Venn diagram or an UpSet plot (when the number of studies is greater than three) to compare the results of each study with the meta-analysis. It also outputs several indicators as described in the description of the tool (see Figure 7). As for the limma tool, annotated expressed genes are displayed in a table which can be ordered and requested.

RNA-seq data meta-analysis example

SMAGEXP was applied to two Recount2 datasets identified with the following IDs : SRP032833 [17] and SRP028180[18]. These two datasets contain human lung squamous cell carcinoma (SCC) data. We first fetch data from these datasets with the recount galaxy tool. Then thanks to the Galaxy DESeq2 tool we launch differential analysis on the following contrasts : invasive versus normal for SRP032833 dataset and tumor versus normal for SRP028180 dataset.

Run a metaRNAseq analysis

The RNA-seq data meta-analysis tool relies on DESeq2 results (see Figure 8).

It outputs a Venn diagram or an UpSet plot (if the number of studies is greater than 2) and the same indicators as in the microarray data analysis tool for both Fisher and inverse normal p-values combinations. It also generates a text file containing summarization of the results of each single analysis and meta-analysis. Potential conflicts between single analyses are indicated by zero values in the "signFC" column 9).

Conclusion

We developed SMAGEXP, a toolsuite dedicated to gene-expression data meta-analysis. This toolsuite proposes quality controls, single analyses and meta-analyses of microarray and RNA-seq data, suggesting appropriate pipelines for each type of data. It delivers fully annnotated results of differentially expressed genes, exportable in several usual formats. Integrated into Galaxy, SMAGEXP is easy to use for biologists and life scientists. R packages metaMA and metaRNAseq thus inherit reproductibility and accessibility support from Galaxy. Furthermore, thanks to Docker, we made these Galaxy tools and their dependencies easy to deploy.

Availability of source code and requirements

- Project name: SMAGEXP
- Project home page: https://github.com/sblanck/smagexp

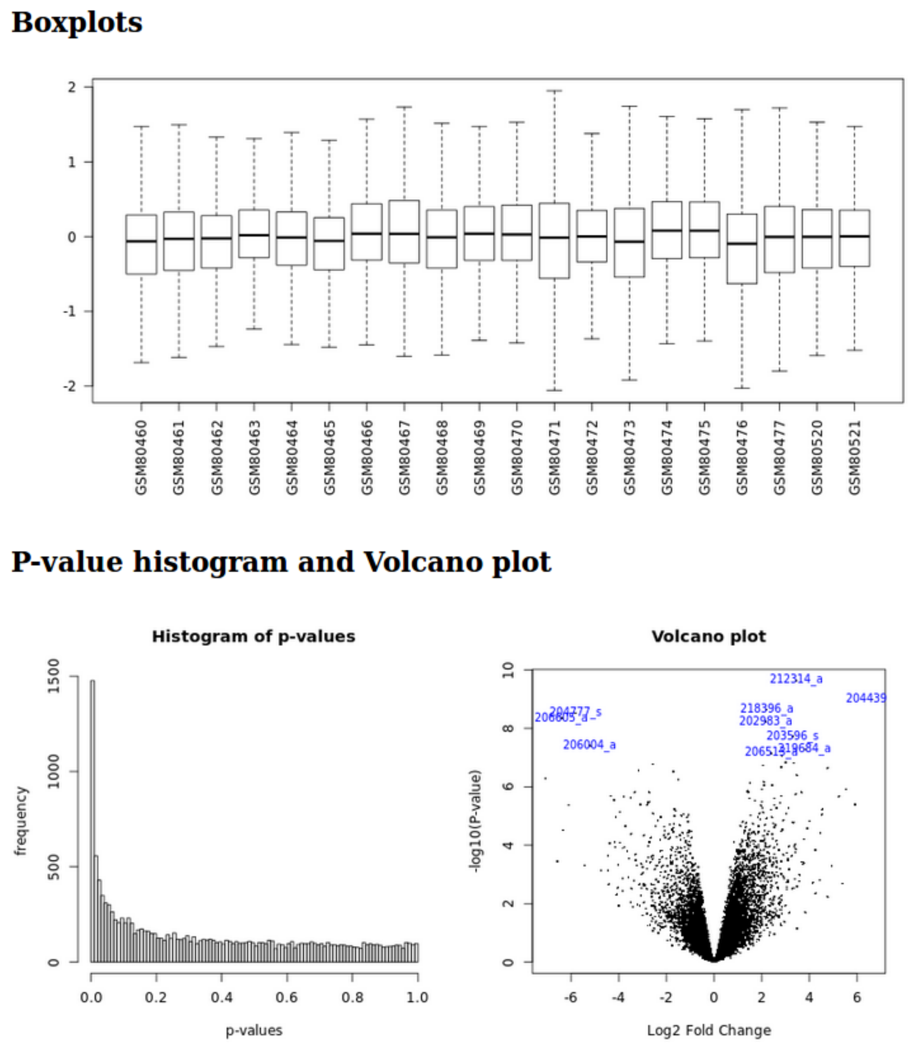

Figure 5. limma analysis tool output plots.

Copy | CSV | Excel — Search:

	ID	adj_P_Val	P_Value	t	B	logFC	Gene_symbol	Gene_title	Gene_ID	Chromosome_annota...	GO_Function_ID
+	212314_at	3.3e-06	2.3e-10	-11.19	13.3288	-3.46	SEL1L3	sel-1 suppressor of lin...	23231	Chromosome 4, NC_00...	
+	204439_at	7.1e-06	1.0e-09	-10.31	12.0465	-6.64	IFI44L	interferon-induced pr...	10964	Chromosome 1, NC_00...	
+	218396_at	1.0e-05	2.3e-09	-9.85	11.3259	-2.20	VPS13C	vacuolar protein sorti...	54832	Chromosome 15, NC_0...	
−	204777_s_at	1.0e-05	2.9e-09	9.71	11.1038	5.81	MAL	mal, T-cell differentiat...	4118	Chromosome 2, NC_00...	GO:0015267///GO:000...
	Gene Symbol: MAL										
	Gene Title: mal, T-cell differentiation protein										
	GO Function ID: GO:0015267, GO:0008289, GO:0016505, GO:0005515, GO:0019911,										
+	206605_at	1.3e-05	4.5e-09	9.47	10.7170	6.39	ENDOU	endonuclease, polyU-s...	8909	Chromosome 12, NC_0...	GO:0003723///GO:000...
+	202983_at	1.4e-05	6.0e-09	-9.32	10.4674	-2.17	HLTF	helicase-like transcrip...	6596	Chromosome 3, NC_00...	GO:0005524///GO:001...
+	203596_s_at	3.9e-05	1.9e-08	-8.70	9.4094	-3.31	IFIT5	interferon-induced pr...	24138	Chromosome 10, NC_0...	GO:0003723///GO:004...
+	206004_at	7.0e-05	4.0e-08	8.33	8.7509	5.21	TGM3	transglutaminase 3	7053	Chromosome 20, NC_0...	GO:0005509///GO:000...
+	219684_at	8.3e-05	5.3e-08	-8.18	8.4819	-3.80	RTP4	receptor (chemosenso...	64108	Chromosome 3, NC_00...	GO:0005515
+	206513_at	1.0e-04	7.1e-08	-8.04	8.2131	-2.39	AIM2	absent in melanoma 2	9447	Chromosome 1, NC_00...	GO:0003690///GO:004...

Show 10 entries

Showing 1 to 10 of 1,000 entries — Previous 1 2 3 4 5 ... 100 Next

Figure 6. limma analysis tool : table of top 10 genes for GSE3524 dataset.

[19]

- Operating system(s): Linux (Galaxy); Platform independent for Galaxy's browser-based user interface.
- Programming language: R
- Other requirements: Galaxy, Docker [20]
- License: MIT License
- Any restrictions to use by non-academics: None
- SciCrunch.org RRID:SCR_016360

SMAGEXP is available on the Galaxy main toolshed [21]. Furthermore, a fully dockerized instance of Galaxy containing SMAGEXP and DESeq2 is available at : https://hub.docker.com/r/sblanck/galaxy-smagexp/

Venn diagram

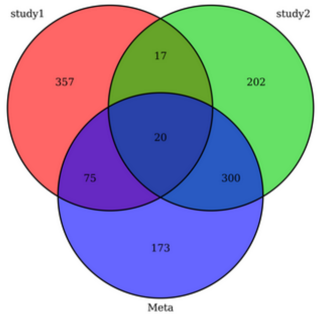

Summary

DE	IDD	Loss	IDR	IRR
568	173	576	30.46	59.32

DE : Number of differentially expressed genes
IDD (Integration Driven discoveries) : number of genes that are declared DE in the meta-analysis that were not identified in any of the individual studies alone
Loss : Number of genes that are identified DE in individual studies but not in meta-analysis
IDR (Integration-driven Discovery Rate) : corresponding proportions of IDD
IRR (Integration-driven Revision) : corresponding proportions of Loss

Figure 7. Venn diagram and summary of microarray data meta-analysis tool results

GeneID	Base mean	log2(FC)	StdErr	Wald-Stats	P-value	P-adj
ENSG00000265190.6	101.667848459903	-5.02994327316764	0.476278688304001	-10.5609245105611	4.52186105012193e-26	1.2756170022394e-21
ENSG00000108576.9	752.853369636084	-5.68489458741731	0.559150886647592	-10.1670134540987	2.78311400192593e-24	3.92558229971652e-20
ENSG00000171004.17	61.0560433708025	4.45947645339064	0.460698196936815	9.67982180751243	3.67355704743372e-22	3.45436814360351e-18
ENSG00000150471.16	92.7223365606503	-3.91012189316814	0.408456528581607	-9.57292054247805	1.03927571285519e-21	7.3294919649112e-18
ENSG00000263155.5	393.952118479798	-3.79214827683006	0.409633385409402	-9.25741995623736	2.09431522607806e-20	1.18161265055324e-16
ENSG00000244734.3	257.773280304236	-3.80955348394829	0.420635151031447	-9.05666935967386	1.3449453378267e-19	6.32348466334854e-16
ENSG00000267881.1	2126.21177987512	4.33697420366533	0.4885788286519	8.87671333535272	6.88649832831602e-19	2.77525882631136e-15
ENSG00000276850.4	94.6312869517772	-4.20194669282102	0.476313487687766	-8.8218091686194	1.12625070263056e-18	3.97144154015102e-15
ENSG00000204305.13	6415.80419271328	-4.17341401530811	0.474415995820123	-8.79695046557932	1.40584327801971e-18	4.40653765254844e-15
ENSG00000137648.17	162.439090763396	3.23640529723304	0.369070732280365	8.76906515246109	1.80155778411351e-18	4.76784441952831e-15
ENSG00000268388.5	647.319788179132	-4.01887899625677	0.458487145724379	-8.76552163726904	1.85913819974518e-18	4.76784441952831e-15
[illegible]	[illegible]	[illegible]	[illegible]	[illegible]	[illegible]	[illegible]

Figure 8. Header of a deseq2 results file

1	2	3	4	5	6	7	8
"ID"	"DE.DE.Results.study.2"	"DE.DE.Results.Study.1"	"DE.DE.fishercomb"	"DE.DE.invnorm"	"FC.Results.study.2"	"FC.Results.Study.1"	"signFC"
"ENSG00000000003.14"	0	0	0	0	1.44392128037606	0.394198152459482	1
"ENSG00000000005.5"	NA	0	0	0	NA	0.353328264055156	NA
"ENSG00000000419.12"	0	0	0	0	2.26611668300573	0.24880345371866	1
"ENSG00000000457.13"	0	0	0	0	0.245905435149477	0.305575355928341	1
"ENSG00000000460.16"	0	0	0	0	-0.106941841356957	0.77811525637832	0
"ENSG00000000938.12"	0	1	1	1	3.61755450768733	-1.92619262759073	0
"ENSG00000000971.15"	0	NA	0	0	-0.429528627278033	-0.11949265196774	-1
"ENSG00000001036.13"	0	0	0	0	-0.0444188345925028	0.0363606559690495	0
"ENSG00000001084.10"	0	0	0	0	-0.0157568902727656	0.380122104918323	0
"ENSG00000001167.14"	0	0	0	0	1.82020228791286	-0.0649996602246056	0
"ENSG00000001460.17"	0	0	0	0	-1.98506186439524	-0.0525169567708359	-1

Figure 9. Header of a metaRNAseq results file

Availability of supporting data and materials

The data sets supporting the microarray meta-analysis example of this article are available in the Gene Expression Omnibus (GEO) database. Their Accession IDs are GSE3524 and GSE13601. The data sets supporting the RNA-seq meta-analysis example of this article are available on Recount2. Their accession IDs are SRP032833 and SRP028180

Documentation, step by step tutorials, examples, galaxy histories and worflow presented in this article are available on github : https://github.com/sblanck/smagexp/tree/master/examples

Declarations

List of abbreviations

- DE : Differentially Expressed
- IDD : Integration Driven discoveries

- GEO : Gene Expression Omnibus
- IDR : Integration-driven Discovery Rate
- IRR : Integration-driven Revision
- NGS : Next Generation Sequencing
- SMAGEXP : Statistical Meta-Analysis for Gene EXPression

Competing Interests

The authors declare that they have no competing interests.

Author's Contributions

The project was initiated by GM who developped metaMA and metaRNASeq R packages. The galaxy tools were developped, installed, documented by SB and tested by SB and GM. The paper was written by SB and GM. All authors read and approved the final manuscript.

References

1. Goecks J, Nekrutenko A, Taylor J, Team TG. Galaxy: a comprehensive approach for supporting accessible, reproducible, and transparent computational research in the life sciences. Genome Biol 2010;11(8):R86.
2. Blankenberg D, Kuster GV, Coraor N, Ananda G, Lazarus R, Mangan M, et al. Galaxy: A Web-Based Genome Analysis Tool for Experimentalists. Current protocols in molecular biology 2010;p. 19–10.
3. Giardine B, Riemer C, Hardison RC, Burhans R, Elnitski L, Shah P, et al. Galaxy: a platform for interactive large-scale genome analysis. Genome research 2005;15(10):1451–1455.
4. Marot G, Foulley JL, Mayer CD, Jaffrezic F. Moderated effect size and P-value combinations for microarray meta-analyses. Bioinformatics 2009;25(20):2692–2699.
5. Hedges L, Olkin I. Statistical Methods for Meta-Analysis. London: Academic Press; 1985.
6. Ritchie ME, Phipson B, Wu D, Hu Y, Law CW, Shi W, et al. limma powers differential expression analyses for RNA-sequencing and microarray studies. Nucleic Acids Research 2015;43(7):e47.
7. Rau A, Marot G, Jaffrézic F. Differential meta-analysis of RNA-seq data from multiple studies. BMC Bioinformatics 2014;15(1):1–10. http://dx.doi.org/10.1186/1471-2105-15-91.
8. Fisher RA. Statistical Methods for Research Workers. Edinburgh: Oliver and Boyd; 1932.
9. Love MI, Huber W, Anders S. Moderated estimation of fold change and dispersion for RNA-seq data with DESeq2. Genome Biology 2014;15:550. http://dx.doi.org/10.1186/s13059-014-0550-8.
10. Edgar R, Domrachev M, Lash AE. Gene Expression Omnibus: NCBI gene expression and hybridization array data repository. Nucleic Acids Res 2002 Jan;30(1):207–210.
11. Davis S, Meltzer P. GEOquery: a bridge between the Gene Expression Omnibus (GEO) and BioConductor. Bioinformatics 2007;14:1846–1847.
12. Huber, W , Carey, J V, Gentleman, R , et al. Orchestrating high-throughput genomic analysis with Bioconductor. Nature Methods 2015;12(2):115–121. http://www.nature.com/nmeth/journal/v12/n2/full/nmeth.3252.html.
13. Conway JR, Lex A, Gehlenborg N. UpSetR: an R package for the visualization of intersecting sets and their properties. Bioinformatics 2017;33(18):2938–2940. http://dx.doi.org/10.1093/bioinformatics/btx364.
14. Collado-Torres L, Nellore A, Kammers K, Ellis SE, Taub MA, Hansen KD, et al. Reproducible RNA-seq analysis using recount2. Nature Biotechnology 2017;http://www.nature.com/nbt/journal/v35/n4/full/nbt.3838.html.
15. Toruner GA, Ulger C, Alkan M, Galante AT, Rinaggio J, Wilk R, et al. Association between gene expression profile and tumor invasion in oral squamous cell carcinoma. Cancer Genet Cytogenet 2004 Oct;154(1):27–35.
16. Estilo CL, O-charoenrat P, Talbot S, Socci ND, Carlson DL, Ghossein R, et al. Oral tongue cancer gene expression profiling: Identification of novel potential prognosticators by oligonucleotide microarray analysis. BMC Cancer 2009 Jan;9:11.
17. Morton ML, Bai X, Merry CR, Linden PA, Khalil AM, Leidner RS, et al. Identification of mRNAs and lincRNAs associated with lung cancer progression using next-generation RNA sequencing from laser micro-dissected archival FFPE tissue specimens. Lung Cancer 2014 Jul;85(1):31–39.
18. Ooi AT, Gower AC, Zhang KX, Vick JL, Hong L, Nagao B, et al. Molecular profiling of premalignant lesions in lung squamous cell carcinomas identifies mechanisms involved in stepwise carcinogenesis. Cancer Prev Res (Phila) 2014 May;7(5):487–495.
19. SMAGEXP; https://github.com/sblanck/smagexp.
20. Galaxy;. https://galaxyproject.org/.
21. Blankenberg D, Von Kuster G, Bouvier E, Baker D, Afgan E, Stoler N, et al. Dissemination of scientific software with Galaxy ToolShed. Genome Biology 2014;15(2):1–3. http://dx.doi.org/10.1186/gb4161.

Click here to access/download
Supplementary Material
article.pdf

Click here to access/download
Supplementary Material
reply to reviewers.pdf
