# Supplementary material for: SMAGEXP: a galaxy tool suite for transcriptomics data meta-analysis
Source: Gigascience. 2019 Jan 29;8(2):giy167. doi: 10.1093/gigascience/giy167 (PMC6354025; doi:10.1093/gigascience/giy167)
Supplement: Response_to_Reviewer_Comments_Original_Submission.pdf [file giy167_response_to_reviewer_comments_original_submission.pdf]

Author's Response To Reviewer Comments

Close

We thank the Editor and the two Reviewers for their comments and suggestions to improve the quality of the paper. We have now taken into account all the points raised in the reviews.

Reply to editor :

SMAGEXP has been registered in the SciCrunch.org database with the RRID SCR:016360. It is included in the Availability of source code and requirements section

Reply to reviewer 1

1. We mentionned it in the results part of the abstract, in the conclusion of the manuscript and in the availability of source code and requirements.

2.a The GEOquery bug is fixed.

2.b We did not find an easy way to include histories inside the docker image. Nevertheless, all the histories, data, and workflow are available on github at https://github.com/sblanck/smagexp/tree/master/examples and can easily, once logged in Galaxy, be imported into the dockerized version of Galaxy.

3. UpSet plots have been added. We chose to keep Venn diagram when the number of studies is <=3 for micro-array and =2 for RNA-seq (since two p-value combination methods are available for this technology). If the number of studies is greater, we plot UpSet diagrams.

4.a A step by step tutorial with screenshots was added in the github documentation

4.b The inline documentation of RNA-seq meta-analysis was added.

4.c This mention was added in the documentation on github.

4.d The .cond file is not needed any more with the import custom data tool. Nevertheless, it is still required for the limma analysis. The format of the .cond file is described in the documentation and examples of .cond file are available on github.

4.e This instruction was added in the documentation.

5. It is not possible to easily retrieve RNA-seq count data with GEOquery from Galaxy. The count files are sometimes available as supplementary data, but the format often varies from one dataset to another one.
We propose to fetch raw count data from recount2 project. We developped a wrapper to retrieve data from recount2 and generate count files. These count files are suitable for the galaxy

DESeq2 tool which was added to the docker image alongside SMAGEXP. Note that recount always outputs the same number of transcripts for a given species. Therefore we have an easy way to meta-analyze rna-seq data from real world.

6. The NAR paper assumes that data coming from micro-array an rna-seq are similar as they calculate their effect sizes the same way. In particular, the calculation described is relevant for Gaussian data, questionnable for count data. As we already mentioned in "Rau et~al.(2014), Differential meta-analysis of RNA-seq data from multiple studies, BMC Bioinformatics 2014;15(1):1--10" , we believe that the Gaussian distribution is not adapted for most RNA-Seq datasets, without transformation. Several transformations have been suggested in the litterature but the automatic choice of the better one is still a question of research for statisticians so we prefer that users treat RNAseq and microarray data seperately, even if they can merge the results via VennDiagrams.

Reply to reviewer 2

1. Docker hub link has been corrected

2. The rdata objects contain Bioconductor ExpressionSet objects, containing expression data and meta-data of the experiments. Their content is described on the github page. They are given for people who are familiar with the R programming language and can be directly loaded in rStudio environment.
It seems difficult to have an interaction between Galaxy and rStudio in the docker container and we believe that people who use Galaxy do not need these rdata inside Galaxy. All the valuable information of these rdata is also available in text format in Galaxy.

3. The workflow and histories are now available on github and can easily be imported in Galaxy (inside the docker container or on a standalone instance)\\
https://github.com/sblanck/smagexp/tree/master/examples

minor :

1. Done

2. Bioconductor citation has been added.

Close
