# Supplementary material for: SMAGEXP: a galaxy tool suite for transcriptomics data meta-analysis
Source: Gigascience. 2019 Jan 29;8(2):giy167. doi: 10.1093/gigascience/giy167 (PMC6354025; doi:10.1093/gigascience/giy167)
Supplement: Response_to_Reviewer_Comments_Revision_1.pdf [file giy167_response_to_reviewer_comments_revision_1.pdf]

Author's Response To Reviewer Comments

Close

We thank Reviewer 1 for his comments and suggestions to improve the quality of the paper. We now answer to all the remaining points raised in this second review :

2.b One small issue -- I found the workflow for the microarray example, but the RNA-seq seems to be missing. Please include this in the repository.
> Unfortunately, the Recount tool is not workflow-friendly. The number of output datasets varies entirely based upon the study. The tool generates as many files as the number of samples in the study with the discover_dataset option. Such discovered datasets cannot be used in workflow, as mentionned at https://planemo.readthedocs.io/en/latest/writing_advanced.html#individual-datasets. That's why we can't publish a workflow for the RNA-seq meta-analysis. Nevertheless the full history of the metaRNAseq analysis is still available on github.

2.b Please also resolve the errors I reported in issue #2 on the SMAGEXP GitHub, and ensure that the workflows provided match up with (and execute successfully within) the dockerised version of SMAGEXP.
> The histories and the worflow are now sync up with the docker image. We added a comment on the issue posted on github : "Generally, this error is due to the fact that the user selects only one .CEL file when running the QCNormalization tool. By selecting several .CEL files when launching the workflow, the error should disappear.
Futhermore, The provided workflow on github should now be synced up with the dockerized version of SMAGEXP."

3. Thank you, this seems like a good solution. Could you please include an example using UpSet plots in the manuscript? This would also serve to illustrate SMAGEXP on a more complicated multi-dataset example.

> The metaRNAseq tutorial has been changed. Now it contains 3 different studies from recount, in order to generate an UpSet Diagram. Example of such a diagram as been added to the manuscript and in the github tutorial.

Close
