# Supplementary material for: SMAGEXP: a galaxy tool suite for transcriptomics data meta-analysis
Source: Gigascience. 2019 Jan 29;8(2):giy167. doi: 10.1093/gigascience/giy167 (PMC6354025; doi:10.1093/gigascience/giy167)
Supplement: Response_to_Reviewer_Comments_Revision_2.pdf [file giy167_response_to_reviewer_comments_revision_2.pdf]

Author's Response To Reviewer Comments

Close

>You need to integrate the GigaDB DOI into the paper, and in the Availability of Supporting Data section you should add a line similar to this:

>Code snapshots and input data is available from the GigaScience GigaDB repository[23].

>Then cite as reference 23.:

>Blanck S; Marot G (2018): Supporting data for "SMAGEXP: a galaxy tool suite for transcriptomics data meta-analysis" GigaScience >Database. http://dx.doi.org/10.5524/100541

The line was added to the manuscript in the Availability of Supporting Data section and cited as reference 23.

> Please also work with our curators to get the DOI live.

I addressed some issues with the curators to get the DOI live but I still don't have accurate information about funding. I will send you the information as soon as i get them.

Close
