# Supplementary material for: SMAGEXP: a galaxy tool suite for transcriptomics data meta-analysis
Source: Gigascience. 2019 Jan 29;8(2):giy167. doi: 10.1093/gigascience/giy167 (PMC6354025; doi:10.1093/gigascience/giy167)
Supplement: Reviewer_1_Report_Original_Submission -- Kieran O'Neill — 3/21/2018 Reviewed [file giy167_reviewer_1_report_original_submission.pdf]

Reviewer Report

Title: SMAGEXP: a galaxy tool suite for transcriptomics data meta-analysis

Version: Original Submission **Date:** 3/21/2018

Reviewer name: Kieran O'Neill

Reviewer Comments to Author:

This tool represents a great initiative towards providing biologists with lower levels of technical expertise with the ability to perform meta-analysis of transcriptomic data. It also has the potential to enable users to provide those analyses in fully-reproducible form as an accompaniment to their manuscripts. Given the abundance of transcriptomic data, these forms of analysis are becoming increasingly common as a step in a biological investigation, and it is important to provide tools to wet lab biologists to be able to do so.

That said, SMAGEXP in its present form has a number of issues, ranging from minor bugs to more major gaps in functionality. For it to have a good chance of being useful and reaching widespread adoption, it will require some additional work, as per my comments below. I thank the authors for their work, and look forward to seeing the revised tool and manuscript.

1. I was pleased to see that you had provided the complete tool and all dependencies as a Docker image. This is extremely valuable both for ease of getting the workflow running (especially for people not familiar with Galaxy), and for enabling fully reproducible analyses to be created and shared. I would love to see that mentioned in the manuscript.

2. However, I was unfortunately not able to actually reproduce your analyses in the paper. GEOQuery failed (see the issue I added to GitHub), while the results of the analysis on the example data sets provided on the GitHub did not match that shown in the paper. To address this, please:

2.a. Fix the bug in GEOQuery

2.b. Provide a version of the Docker image containing all of the data and analyses from the paper pre-loaded. I believe Galaxy allows for histories to be saved, which would be one way of doing this.

3. The meta-analyses at present create Venn diagrams. However, Venn diagrams struggle with four sets, and don't work at all beyond that. Please provide the option of a set intersection visualisation that will scale to more than three datasets. For this purpose, I would recommend UpSet plots, which are implemented in the UpSetR package in Bioconductor.

4. There is some documentation on the GitHub readme, and inline documentation on most of the tools, which is good. However, since the target users are meant to be non-technical, more detailed documentation would be essential for this tool's uptake.

4.a. A step by step tutorial of all the functionality provided, with either screenshots or a video would be good.

4.b. The RNA-seq meta-analysis tool has no inline documentation; this should be provided.

4.c. It would be useful, in the "import custom data" tool, to mention that data should first be loaded via Galaxy's Get Data > Upload File.

4.d. It would also be useful to mention how data can be imported in the absence of a condition file (as is

the case for the example data provided on the GitHub).
4.e. In the readme instructions for running the Docker container, a sentence instructing the user to then open a web browser and type in localhost:8080 would be helpful. (As an advanced user I could deduce this, but this may not be obvious to the target users.)
5. For the RNA-seq meta-analysis, there is the text "Must have the same number of row in each study." Given the wide variety of gene models and genome versions that RNA-seq data sets have been aligned to over the past decade, this seems problematic. The RNA-seq example provided in the paper also only operates on the carefully pre-generated example files, which have the same number of rows and row names (tagNNNN). This is not really a fair representation of a pair of RNA-seq data sets that a user may want to perform a meta-analysis on.
Can you please provide a real-world example of how SMAGEXP operates on RNA-seq data from different data sets? I believe there are existing Galaxy tools which can interconvert between identifiers and filter or join data sets. Providing the complete saved Galaxy workflow for this would also be extremely helpful for users trying to use this tool in future. It would also be useful for there to be a version of GEOQuery which can retrieve RNA-seq data.
6. Not addressed in this paper is the question of meta-analyses that combine microarray and RNA-seq data. An example of how this can be done is given in a recent NAR paper, and their results were validated using in vivo using immunohistochemistry. https://doi.org/10.1093/nar/gkx714 Could you please either provide this functionality as part of SMAGEXP, or discuss why it was not included, and the steps towards including it.

Level of Interest

Please indicate how interesting you found the manuscript: Choose an item.

Quality of Written English

Please indicate the quality of language in the manuscript: Needs some language corrections before being published

Declaration of Competing Interests

Please complete a declaration of competing interests, considering the following questions:

- Have you in the past five years received reimbursements, fees, funding, or salary from an organisation that may in any way gain or lose financially from the publication of this manuscript, either now or in the future?
- Do you hold any stocks or shares in an organisation that may in any way gain or lose financially from the publication of this manuscript, either now or in the future?
- Do you hold or are you currently applying for any patents relating to the content of the manuscript?

- Have you received reimbursements, fees, funding, or salary from an organization that holds or has applied for patents relating to the content of the manuscript?
- Do you have any other financial competing interests?
- Do you have any non-financial competing interests in relation to this paper?

If you can answer no to all of the above, write 'I declare that I have no competing interests' below. If your reply is yes to any, please give details below.

I declare that I have no competing interests' below

I agree to the open peer review policy of the journal. I understand that my name will be included on my report to the authors and, if the manuscript is accepted for publication, my named report including any attachments I upload will be posted on the website along with the authors' responses. I agree for my report to be made available under an Open Access Creative Commons CC-BY license (http://creativecommons.org/licenses/by/4.0/). I understand that any comments which I do not wish to be included in my named report can be included as confidential comments to the editors, which will not be published.

I agree to the open peer review policy of the journal

To further support our reviewers, we have joined with Publons, where you can gain additional credit to further highlight your hard work (see: https://publons.com/journal/530/gigascience). On publication of this paper, your review will be automatically added to Publons, you can then choose whether or not to claim your Publons credit. I understand this statement.

Yes Choose an item.
