# Supplementary material for: SMAGEXP: a galaxy tool suite for transcriptomics data meta-analysis
Source: Gigascience. 2019 Jan 29;8(2):giy167. doi: 10.1093/gigascience/giy167 (PMC6354025; doi:10.1093/gigascience/giy167)
Supplement: Reviewer_1_Report_Revision_1 -- Kieran O'Neill — 7/25/2018 Reviewed [file giy167_reviewer_1_report_revision_1.pdf]

Reviewer Report

Title: SMAGEXP: a galaxy tool suite for transcriptomics data meta-analysis

Version: Revision 1 **Date:** 7/25/2018

Reviewer name: Kieran O'Neill

Reviewer Comments to Author:

The tool and manuscript are both greatly improved since the last version, and I appreciate the authors' having addressed most of my concerns. I still have have a few remaining, as noted inline below and summarised here: Please ensure that the examples work with the dockerised version of SMAGEXP, and that workflow files are provided in addition to the histories. I'd also like to see an example of the UpSet plot functionality in action. I believe these should be fairly minor, and that the paper should be ready to publish once they are addressed.
Reply to reviewer 1
1. We mentionned it in the results part of the abstract, in the conclusion of the manuscript and in the availability of source code and requirements.
> Thank you.
2.a The GEOquery bug is fixed.
> Thank you. GEOquery seems to now be working.
2.b We did not find an easy way to include histories inside the docker image. Nevertheless, all the histories, data, and workflow are available on github at https://github.com/sblanck/smagexp/tree/master/examples and can easily, once logged in Galaxy, be imported into the dockerized version of Galaxy.
> Thank you, this seems like an adequate solution.
> One small issue -- I found the workflow for the microarray example, but the RNA-seq seems to be missing. Please include this in the repository.
> Please also resolve the errors I reported in issue #2 on the SMAGEXP GitHub, and ensure that the workflows provided match up with (and execute successfully within) the dockerised version of SMAGEXP.
3. UpSet plots have been added. We chose to keep Venn diagram when the number of studies is <=3 for micro-array and =2 for RNA-seq (since two p-value combination methods are available for this technology). If the number of studies is greater, we plot UpSet diagrams.
> Thank you, this seems like a good solution. Could you please include an example using UpSet plots in the manuscript? This would also serve to illustrate SMAGEXP on a more complicated multi-dataset example.
4.a A step by step tutorial with screenshots was added in the github documentation
> Thank you. This is excellent and appreciated.
4.b The inline documentation of RNA-seq meta-analysis was added.
> Thank you, the documentation is much improved.
4.c This mention was added in the documentation on github.

> Thank you.
4.d The .cond file is not needed any more with the import custom data tool. Nevertheless, it is still required for the limma analysis. The format of the .cond file is described in the documentation and examples of .cond file are available on github.
> Thank you.
4.e This instruction was added in the documentation.
> Great, thank you.
5. It is not possible to easily retrieve RNA-seq count data with GEOquery from Galaxy. The count files are sometimes available as supplementary data, but the format often varies from one dataset to another one.
We propose to fetch raw count data from recount2 project. We developped a wrapper to retrieve data from recount2 and generate count files. These count files are suitable for the galaxy DESeq2 tool which was added to the docker image alongside SMAGEXP. Note that recount always outputs the same number of transcripts for a given species. Therefore we have an easy way to meta-analyze rna-seq data from real world.
> This seems like a great solution to the issue I raised. Thank you.
6. The NAR paper assumes that data coming from micro-array an rna-seq are similar as they calculate their effect sizes the same way. In particular, the calculation described is relevant for Gaussian data, questionnable for count data. As we already mentioned in "Rau et~al.(2014), Differential meta-analysis of RNA-seq data from multiple studies, BMC Bioinformatics 2014;15(1):1--10" , we believe that the Gaussian distribution is not adapted for most RNA-Seq datasets, without transformation. Several transformations have been suggested in the litterature but the automatic choice of the better one is still a question of research for statisticians so we prefer that users treat RNAseq and microarray data seperately, even if they can merge the results via VennDiagrams.
> I guess this is acceptable.

Level of Interest

Please indicate how interesting you found the manuscript: Choose an item.

Quality of Written English

Please indicate the quality of language in the manuscript: Choose an item.

Declaration of Competing Interests

Please complete a declaration of competing interests, considering the following questions:

- Have you in the past five years received reimbursements, fees, funding, or salary from an organisation that may in any way gain or lose financially from the publication of this manuscript, either now or in the future?

- Do you hold any stocks or shares in an organisation that may in any way gain or lose financially from the publication of this manuscript, either now or in the future?
- Do you hold or are you currently applying for any patents relating to the content of the manuscript?
- Have you received reimbursements, fees, funding, or salary from an organization that holds or has applied for patents relating to the content of the manuscript?
- Do you have any other financial competing interests?
- Do you have any non-financial competing interests in relation to this paper?

If you can answer no to all of the above, write 'I declare that I have no competing interests' below. If your reply is yes to any, please give details below.

I declare that I have no competing interests.

I agree to the open peer review policy of the journal. I understand that my name will be included on my report to the authors and, if the manuscript is accepted for publication, my named report including any attachments I upload will be posted on the website along with the authors' responses. I agree for my report to be made available under an Open Access Creative Commons CC-BY license (http://creativecommons.org/licenses/by/4.0/). I understand that any comments which I do not wish to be included in my named report can be included as confidential comments to the editors, which will not be published.

I agree to the open peer review policy of the journal

To further support our reviewers, we have joined with Publons, where you can gain additional credit to further highlight your hard work (see: https://publons.com/journal/530/gigascience). On publication of this paper, your review will be automatically added to Publons, you can then choose whether or not to claim your Publons credit. I understand this statement.

Yes Choose an item.
