# Supplementary material for: SMAGEXP: a galaxy tool suite for transcriptomics data meta-analysis
Source: Gigascience. 2019 Jan 29;8(2):giy167. doi: 10.1093/gigascience/giy167 (PMC6354025; doi:10.1093/gigascience/giy167)
Supplement: Reviewer_2_Report_Original_Submission -- Nitesh Turaga — 3/29/2018 Reviewed [file giy167_reviewer_2_report_original_submission.pdf]

Reviewer Report

Title: SMAGEXP: a galaxy tool suite for transcriptomics data meta-analysis

Version: Original Submission **Date:** 3/29/2018

Reviewer name: Nitesh Turaga

Reviewer Comments to Author:

The paper is well written and wraps two R packages for Galaxy.
Table 1 is very helpful for users. It would be useful for each tool wrapped in Galaxy to have a label "Inputs" in the documentation.
1. Docker hub link is broken-https://hub.docker.com/r/sblanck/galaxy-SMAGEXP/. (line 56, page 5)
2. The intermediate steps stored as RData galaxy datatypes seem to be a black box, and the user cannot access them without pulling them onto their own machine or using an interactive environment within Galaxy. It would be useful to show how to access it within an interactive environment (Visualization - Juypter notebook). A more detailed description of what the contents in the intermediate RData object(s) is useful as well.
3. The docker container can also host an example workflow of the usage.
Minor:
1. Typos need to be checked throughout, within the figures and tables as well.
2. Considering the Bioconductor infrastructure used within the R scripts, it seems it would be appropriate to cite Bioconductor.

Level of Interest

Please indicate how interesting you found the manuscript: Choose an item.

Quality of Written English

Please indicate the quality of language in the manuscript: Needs some language corrections before being published

Declaration of Competing Interests

Please complete a declaration of competing interests, considering the following questions:

- Have you in the past five years received reimbursements, fees, funding, or salary from an organisation that may in any way gain or lose financially from the publication of this manuscript, either now or in the future?
- Do you hold any stocks or shares in an organisation that may in any way gain or lose financially from the publication of this manuscript, either now or in the future?

- Do you hold or are you currently applying for any patents relating to the content of the manuscript?
- Have you received reimbursements, fees, funding, or salary from an organization that holds or has applied for patents relating to the content of the manuscript?
- Do you have any other financial competing interests?
- Do you have any non-financial competing interests in relation to this paper?

If you can answer no to all of the above, write 'I declare that I have no competing interests' below. If your reply is yes to any, please give details below.

I declare that I have no competing interests

I agree to the open peer review policy of the journal. I understand that my name will be included on my report to the authors and, if the manuscript is accepted for publication, my named report including any attachments I upload will be posted on the website along with the authors' responses. I agree for my report to be made available under an Open Access Creative Commons CC-BY license (http://creativecommons.org/licenses/by/4.0/). I understand that any comments which I do not wish to be included in my named report can be included as confidential comments to the editors, which will not be published.

I agree to the open peer review policy of the journal

To further support our reviewers, we have joined with Publons, where you can gain additional credit to further highlight your hard work (see: https://publons.com/journal/530/gigascience). On publication of this paper, your review will be automatically added to Publons, you can then choose whether or not to claim your Publons credit. I understand this statement.

Yes Choose an item.
